# Andere spezifische Diabetesformen und exokrine Pankreasinsuffizienz (Update 2023)

**DOI:** 10.1007/s00508-022-02123-x

**Published:** 2023-04-20

**Authors:** Susanne Kaser, Sabine E. Hofer, Lili Kazemi-Shirazi, Andreas Festa, Yvonne Winhofer, Harald Sourij, Helmut Brath, Michaela Riedl, Michael Resl, Martin Clodi, Thomas Stulnig, Claudia Ress, Anton Luger

**Affiliations:** 1 Universitätsklinik für Innere Medizin 1, Medizinische Universität Innsbruck, Anichstraße 35, 6020 Innsbruck, Österreich; 2 Universitätsklinik für Pädiatrie 1, Medizinische Universität Innsbruck, Innsbruck, Österreich; 3 Klinische Abteilung für Gastroenterologie und Hepatologie, Universitätsklinik für Innere Medizin III, Medizinische Universität Wien, Wien, Österreich; 4 Abteilung für Innere Medizin I, LK Stockerau, Stockerau, Österreich; 5 Klinische Abteilung für Endokrinologie und Stoffwechsel, Universitätsklinik für Innere Medizin III, Medizinische Universität Wien, Wien, Österreich; 6 Klinische Abteilung für Endokrinologie und Diabetologie, Universitätsklinik für Innere Medizin, Medizinische Universität Graz, Graz, Österreich; 7 Mein Gesundheitszentrum Favoriten, Österreichische Gesundheitskasse, Wien, Österreich; 8 Abteilung für Innere Medizin, Konventhospital der Barmherzigen Brüder Linz, Linz, Österreich; 9 ICMR – Institute for Cardiovascular and Metabolic Research, JKU Linz, Linz, Österreich; 10 3. Medizinische Abteilung und Karl Landsteiner Institut für Stoffwechselerkrankungen und Nephrologie, Klinik Hietzing, Wien, Österreich

**Keywords:** Spezifische Diabetesformen, Exokrine Pankreasinsuffizienz, Genetische Diabetesformen, Medikamentös-induzierte Diabetesformen, Diabetes im Rahmen anderer endokrinologischer Erkrankungen, Other specific diabetes types, Exocrine pancreatic insufficiency, Genetic diabetes forms, Drug induced diabetes, Diabetes due to other endocrine disorders

## Abstract

Die unter der Kategorie „andere spezifische Diabetesformen“ zusammengefassten Störungen des Glukosestoffwechsels stellen pathophysiologisch und therapeutisch eine sehr heterogene Krankheitsgruppe dar. Umfasst werden Diabetesformen, die im Rahmen von anderen endokrinologischen Erkrankungen auftreten (z. B. Akromegalie, Cushing-Syndrom), medikamentös induzierte Diabetesformen (z. B. Antipsychotikatherapie, Glukokortikoidtherapie, HAART, Checkpoint-Inhibitoren, genetische Formen (z. B. i. R. eines MODY, neonataler Diabetes, Down-Syndrom, Klinefelter-Syndrom, Turner-Syndrom), pankreoprive Formen (z. B. postoperativ, Pankreatitis, Pankreastumoren, Hämochromatose, zystische Fibrose), Infektionen (z. B. kongenitale Rötelninfektion) und seltene autoimmune Formen (z. B. Stiffman-Syndrom). Die Diagnose der spezifischen Diabetesform kann die therapeutischen Erwägungen beeinflussen. Nicht nur pankreoprive Formen, sondern auch Typ 1 oder langjähriger Typ 2 Diabetes mellitus sind häufig mit einer exokrinen Pankreasinsuffizienz assoziiert.

## Abstract (en)

The heterogenous category “specific types of diabetes due to other causes” encompasses disturbances in glucose metabolism due to other endocrine disorders such as acromegaly or hypercortisolism, drug-induced diabetes (e.g. antipsychotic medications, glucocorticoids, immunosuppressive agents, highly active antiretroviral therapy (HAART), checkpoint inhibitors), genetic forms of diabetes (e.g. Maturity Onset Diabetes of the Young (MODY), neonatal diabetes, Down‑, Klinefelter- and Turner Syndrome), pancreatogenic diabetes (e.g. postoperatively, pancreatitis, pancreatic cancer, haemochromatosis, cystic fibrosis), and some rare autoimmune or infectious forms of diabetes. Diagnosis of specific diabetes types might influence therapeutic considerations. Exocrine pancreatic insufficiency is not only found in patients with pancreatogenic diabetes but is also frequently seen in type 1 and long-standing type 2 diabetes.

## Diabetesformen im Rahmen anderer endokriner Erkrankungen

Endokrinologische Erkrankungen, die mit Glukosestoffwechselstörungen einhergehen und deren diagnostische Tests sind in Tab. 1 dargestellt.

**Table 1:** 

Erkrankung	Screening und Diagnostik
Cushing Syndrom	24 h Harn Cortisol, Serum oder Speichel Mitternachtscortisol, 1 mg Dexamethason Suppressionstest
Akromegalie	Serum IGF‑1, 75 g OGTT: GH Nadir
Hyperthyreose	TSH, fT4, fT3
Phäochromozytom	Metanephrin und Normetanephrin im Serum oder 24 h Sammelharn
Primärer Hyperaldosteronismus	Renin/Aldosteron Ratio
Glukagonom	Plasma Glukagon
Somatostatinom	Plasma Somatostatin

*IGF‑1* Insulin like growth factor‑1, *OGTT* oraler Glukosetoleranztest, *TSH* Thyreotropin

### Cushing-Syndrom

In der überwiegenden Mehrzahl ist ein Cushing-Syndrom auf eine vermehrte ACTH-Produktion zurückzuführen. Diese wiederum tritt am häufigsten bei ACTH-sezernierenden Hypophysenadenomen auf, seltener sind ektope ACTH-Quellen bei meist malignen Tumoren (z. B. Bronchialkarzinom, Thymuskarzinoid). Die häufigste Ursache für ACTH-unabhängige Cushing Syndrome sind Nebennierenadenome, seltener Nebennierenkarzinome oder mikro- oder makronoduläre adrenale Hyperplasien [3]. Die klinischen Symptome ähneln teils den diagnostischen Kriterien eines metabolischen Syndroms. Neben einer Gewichtszunahme, einer gestörten Glukosetoleranz bis zum Auftreten eines Diabetes mellitus und einer arteriellen Hypertonie finden sich häufig osteoporotische Wirbelkörperfrakturen, ein Hypogonadismus und spezifische Hautveränderungen, insbesondere rote Striae (Striae rubrae), eine Myopathie und auch psychiatrische Auffälligkeiten [3]. Die Therapie erfolgt sofern möglich chirurgisch, je nach Ätiologie finden ansonsten Dopamin-Agonisten oder das Somatostatinanalogon Pasireotid (Hypophysenadenome) oder adrenostatisch bzw. adrenolytisch wirkende Medikamente (ektope oder adrenale Formen) bzw. strahlentherapeutische Verfahren Anwendung (hypophysäre Formen) [3]. Bezüglich therapeutischer Überlegungen in Zusammenhang mit der Glukosestoffwechselstörung s. medikamentös-induzierte Diabetesformen.

### Akromegalie

Zum ganz überwiegenden Teil ist eine überschießende Sekretion von Wachstumshormon bei Hypophysenadenomen Ursache einer Akromegalie, nur sehr selten ist eine ektope GHRH-Sekretion für eine Akromegalie verantwortlich. Neben unspezifischen Symptomen wie Cephalea, Hyperhidrosis, Schlafapnoe, Hypertonie und Arthralgien sind akrales Wachstum und Makroglossie charakteristische Symptome einer Akromegalie. Begleitet werden diese von metabolischen Komplikationen in Form einer Glukosetoleranzstörung bzw. eines manifesten Diabetes mellitus sowie einer Dyslipidämie [4]. Die Serum IGF‑1 Konzentration wird zum Screening herangezogen, wobei ein präexistenter Diabetes mellitus ebenso wie katabole Zustandsbilder oder eine eingeschränkte Leberfunktion zu falsch negativen Ergebnissen führen können. Bestätigt wird das Vorliegen einer Akromegalie mittels eines 75 g oralen Glukosetoleranztests (OGTT) (humanes Wachstumshormon (GH) Nadir > 0,4 ug/l). Bei unklaren diagnostischen Konstellationen kann bei Patient:innen mit diabetischer Stoffwechsellage ein dynamischer Test mit Galanin oder Thyreotropin-Releasing-Hormone erwogen werden [5].

Therapeutisch ist eine chirurgische Sanierung anzustreben, sollte diese nicht möglich bzw. nicht erfolgreich sein, kommen bei milden Formen Dopaminagonisten, üblicherweise jedoch Somatostatinanaloga oder der GH-Rezeptor-Antagonist Pegvisomant zum Einsatz, ggf. auch strahlentherapeutische Verfahren [4, 6].

Pathophysiologisch führt eine kontinuierliche exzessive Erhöhung der GH-Konzentration zu einer Störung im Insulinsignaltransduktionsweg klinisch einer Insulinresistenz entsprechend. Die Therapie eines Akromegalie-assoziierten Diabetes unterscheidet sich prinzipiell nicht von der eines Diabetes mellitus Typ 2 [5].

### Andere mit DM-assoziierte Endokrinopathien

Ein primärer Hyperaldosteronismus, ebenso wie ein Phäochromozytom, eine manifeste Hyperthyreose oder die seltenen neuroendokrinen Neoplasien Glukagonom und Somatostatinom können mit Störungen des Glukosestoffwechsels einhergehen (Tab. 1).

## Medikamentös-induzierte Diabetesformen

Zahlreiche Medikamente führen in unterschiedlicher Ausprägung zu einer Beeinträchtigung des Glukosestoffwechsels, wichtige Vertreter sind in Tab. 2 zusammengefasst.

**Table 2:** 

Antiinfektiva	Fluoroquinolone (Moxifloxacin)
	Antiretrovirale HIV-Therapie (Proteaseinhibitoren, NRTIs)
	Pentamidin
Antipsychotika	Erstgenerationsantipsychotika (Chlorpromazin, Perphenazin, andere Phenothiazine)
	Zweitgenerationsantipsychotika (Clozapin, Iloperidon, Olanzapin, Paliperidon, Quetiapin, Risperidon)
Anithypertensiva	Betablocker (Atenolol, Metoprolol, Propranolol)
	Thiaziddiuretika (Hydrochlorothiazid, Chlorthalidon, Chlorothiazid, Indapamid)
Vasopressoren	Epinephrin, Norepinephrin
Hormone	Glukokortikoide
	Orale Kontrazeptiva (Östrogen-Progestagen Kombinationspräparate, Progestin Monopräparate)
	Progestin (Megestrolacetat)
	Wachstumshormon, Tesamorelin
	Thyroxin
Lipidsenker	Nikotinsäurederivate, Statine
Immunsuppressiva	Tacrolimus, Cyclosporin A, Sirolimus
Antikörper	Pembrolizumab, Nivolumab, Ipilimumab
Andere	Somatostatinanaloga, Diazoxid, IFN‑γ

Neben Glukokortikoiden sind insbesondere verschiedene typische, aber auch atypische Antipsychotika – unter anderem bedingt durch eine gesteigerte Nahrungszufuhr und entsprechender Gewichtszunahme – mit einem erhöhten Diabetesrisiko verbunden (siehe Kapitel Abrahamian et al., Psychische Erkrankungen und Diabetes mellitus) [17].

Jegliche systemische Glukokortikoidtherapie ist mit einer erhöhten Diabetesinzidenz verbunden. Eine nur kurzzeitige, meist hochdosierte Glukokortikoidtherapie kann zu einer transienten Hyperglykämie führen, während eine längerdauernde Glukokortikoidtherapie u. a. durch eine Gewichtszunahme auch zu einer persistierenden Hyperglykämie führen kann [18]. Das Risiko für das Auftreten einer Hyperglykämie ist vor allem bei > 65-jährigen Patient:innen, zugrundeliegender Glukosestoffwechselstörung, reduzierter Nierenfunktion und rheumatologischen bzw. nephrologischen Patient:innen in Abhängigkeit von der Glukokortikoiddosis und Therapiedauer signifikant erhöht [19, 20]. Charakterisiert ist ein Glukokortikoid-induzierter Diabetes bei morgendlicher Glukokortikoidgabe durch eine meist normale Nüchternglukosekonzentration und eine ausgeprägte Hyperglykämie untertags. Infolgedessen sind postprandiale Glukosemessungen zum Screening und zur Monitorisierung besser geeignet als alleinige Nüchternglukosemessungen [21]. Der HbA1c Wert eignet sich nur bei längerfristiger chronischer Glukokortikoidtherapie zur Therapieüberwachung.

Die diabetogene Stoffwechsellage ist bei Hyperkortisolismus neben einer meist ausgeprägten Gewichtszunahme auch auf eine hepatische und periphere Insulinresistenz sowie eine verminderte Insulinsekretion zurückzuführen [22]. Die Behandlung unterscheidet sich nicht von der eines Diabetes mellitus Typ 2 [23]. Sie richtet sich nach Pharmakokinetik des verwendeten Glukokortikoidpräparates und dem daraus resultierenden Blutzuckertagesprofil sowie den Komorbiditäten der Patient:innen. Als Ziel, insbesondere im stationären Bereich, gilt es, einen Blutzucker von < 180 mg/dl zu erreichen.

Eine besondere Diabetesform stellt der sogenannte Posttransplantationsdiabetes (NODAT: new onset of diabetes after transplantation) dar. Die Prävalenz wird abhängig von dem transplantierten Organ langfristig mit bis zu 40 % angegeben [24, 25]. Neben Glukokortikoiden weisen vor allem Calcineurin Inhibitoren (insbesondere Tacrolimus) ein diabetogenes Potenzial auf. Die Behandlung des NODAT unterscheidet sich zwar grundsätzlich nicht von der eines Diabetes mellitus Typ 2, die Therapieoptionen sind jedoch häufig aufgrund der Komorbiditäten eingeschränkt.

HAART (insbesondere Proteaseinhibitoren und Nukleosidische Reverse-Transkriptase-Inhibitoren (NRTIs)) kann gemeinsam mit der Inflammation einer chronischen HIV-Infektion diabetogen wirken. So zeigte eine rezente Arbeit an 522 österreichischen Patient:innen mit chronischer HIV-Infektion, dass 50 % der im Schnitt 42-jährigen Teilnehmer:innen insulinresistent waren [26]. Zusätzlich zeigte sie ein geringes Bewusstsein für metabolische Erkrankungen in der Therapie dieser vulnerablen Gruppen: 60 % der Diabetesfälle waren nicht diagnostiziert, nur 13 % der Patient:innen mit Dyslipidämie waren therapiert. Die Wahl metabolisch neutraler antiretroviraler Medikation, regelmäßige Kontrollen metabolischer Parameter und ggf. frühzeitige Therapie scheinen ratsam (s. bitte Absatz Insulin-Resistenz und Diabetes bei Lipodystrophie Syndromen).

Antikörper wie Pembrolizumab, Nivolumab und Ipilimumab, eingesetzt in der Tumortherapie, können zu einer β‑Zell-Zerstörung in Form eines Autoimmundiabetes führen [7–9].

In der Gruppe der Antihypertensiva weisen vor allem Thiaziddiuretika und nicht-vasodilatierend wirkende Betablocker ein mildes diabetogenes Potenzial auf [27]. Eine Statintherapie ist vor allem bei Hochrisikopatient:innen mit einer erhöhten Diabetesinzidenz verbunden, wobei der kardiovaskuläre Benefit dem metabolischen Risiko um ein Vielfaches überlegen ist [28]. Im Schnitt ist der HbA1c Wert bei Personen mit Diabetes um 0,14 % höher, der Effekt scheint bei Männern ausgeprägter zu sein als bei Frauen [29].

## Insulin-Resistenz und Diabetes bei Lipodystrophie Syndromen

Die Lipodystrophie Syndrome umfassen eine heterogene Gruppe von seltenen kongenitalen und erworbenen Erkrankungen, die durch einen generalisierten oder partiellenVerlust von Fettgewebe charakterisiert sind, der nicht auf Mangelernährung zurückzuführen ist [30–32]. Sie sind mit einer Reihe von metabolischen Komplikationen wie Insulin-Resistenz, Diabetes mellitus, Hypertriglyzeridämie und Steatosis hepatis vergesellschaftet, häufig auch mit Akanthosis nigricans, polyzystischem Ovar-Syndrom und eruptiven Xanthomen. Die Ausprägung der Komorbiditäten variiert bei den verschiedenen Subtypen und ist zum Teil auf die infolge dysfuktioneller Fettzellen ektope Fettspeicherung in Leber, Muskeln und anderen Organen zurückzuführen. Hormone des Fettgewebes, allen voran Leptin, sind reduziert. Dies trägt zu den metabolischen Störungen und zur häufig assoziierten Hyperphagie bei.

Die zwei häufigsten genetischen Formen sind die autosomal rezessiv vererbte kongenitale generalisierte Lipodystrophie und die autosomal dominant vererbte familiäre partielle Lipodystrophie mit jeweils mehreren Subtypen [30–32]. Weitere Formen sind die erworbene generalisierte Lipodystrophie, die erworbene partielle Lipodystrophie und die Progeria assoziierte Lipodystrophie. Die Prävalenz von genetischen Lipodystrophien wird auf 1 pro Million Menschen geschätzt, die Zahl der undiagnostizierten Erkrankungen ist hoch [30]. Die durch die antiretrovirale Therapie mit Protease-Inhibitoren und nukleosidischen Reverse-Transkriptase-Inhibitoren bei HIV-Patient:innen induzierte Lipodystrophie ist die häufigste Variante der erworbenen Lipodystrophien, tritt mit neuen Therapien aber nun seltener auf [33].

Die Therapie hat die Besserung bzw. Prävention der mit Lipodystrophie assoziierten Komorbiditäten zum Ziel. Neben der Lebenstil-Modifikation sind das in erster Linie Metformin und Fibrate, andere orale Antidiabetika und Insulin, Omega-3-Fettsäuren sowie das Leptin-Analogon Metreleptin [31, 32]. Darüber hinaus sind noch eine Reihe anderer Pharmaka insbesondere zur Reduktion der erhöhten Triglyzerid-Konzentrationen in klinischer Erprobung [32].

## Genetische Diabetesformen

Unter genetischen Formen wird ein heterogenes Krankheitsbild zusammengefasst. Am häufigsten handelt es sich um erblich bedingte Diabetesformen, zurückzuführen auf eine β‑Zell-Dysfunktion (MODY) oder aber eine Glukosestoffwechselstörung im Rahmen von anderen genetischen Erkrankungen (s. bitte auch Absatz Insulin-Resistenz und Diabetes bei Lipodystrophie Syndromen). Selten sind genetische Störungen der Insulinwirkung (Tab. 3).

**Table 3:** 

Genetische Störungen der Betazellfunktion	MODY
	Transienter oder permanenter neonataler Diabetes
	Mitochondriale Diabetesformen
	Andere
Genetische Störungen der Insulinwirkung	Typ A Insulinresistenz
	Leprechaunismus
	Rabson-Mendenhall-Syndrom
	Lipoatropher Diabetes
	Andere
Mit Diabetes mellitus assoziierte andere genetische Erkrankungen	Down-Syndrom
	Klinefelter-Syndrom
	Turner-Syndrom
	Wolfram-Syndrom
	Friedreich-Ataxie
	Chorea Huntington
	Laurence-Moon-Biedl-Syndrom
	Dystrophia myotonica
	Porphyrie
	Prader-Willi-Syndrom
	Andere

### MODY (Maturity-onset diabetes of the young)

Die durchaus nicht selbsterklärende Bezeichnung „Maturity-onset diabetes of the young“ erfordert den historischen Kontext; sie beruht auf der in den 1960-er Jahren strikt geltenden Annahme zweier Diabetesformen, einer im jungen Alter auftretenden Form, die der Insulintherapie bedarf (heute: Typ 1 Diabetes) und einer „milden“ Diabetesform, die typischerweise im Erwachsenenalter auftritt (heute: Typ 2 Diabetes) [34]. Im Jahre 1974 erfolgte anhand der Stammbäume dreier Familien in London die umfassende Beschreibung einer familiären, „milden“ Diabetesform mit dominantem Erbgang, die auch bereits im Kindes- und jungen Erwachsenenalter zu diagnostizieren war [35].

Nach heutigem Wissen erscheint die Bezeichnung „monogenetischer“ Diabetes (gegenüber der historischen Bezeichnung „Maturity-onset diabetes of the young“) sinnvoll. Weiters rückt die aktuelle Literatur von der ursprünglichen numerischen Bezeichnung der MODY Subtypen ab; so wird etwa die vormals als MODY 3 bezeichnete Form heute – der zugrundeliegenden Mutation entsprechend – als HNF1A-MODY bezeichnet [36].

#### Epidemiologie

MODY gehört zu den seltenen Diabetesformen, eine häufig in der Literatur genannte Zahl beziffert die Prävalenz als < 5 % aller Diabetesfälle [36]. Die Literatur hierzu ist allerdings lückenhaft, so wurden etwa die meisten Studien bei weißen Europäer:innen durchgeführt, wonach ethnische Unterschiede, die anzunehmen sind, kaum erforscht sind. Die Prävalenz wird mit 1/23.000 bei Kindern und 1/10.000 bei jungen Erwachsenen angegeben [37–39]. Ungeachtet der tatsächlichen Prävalenz ist eine hohe Dunkelziffer im Sinne einer Unterdiagnose anzunehmen [37, 38, 40]. Studien aus UK legen nahe, dass mehr als 80 % der Fälle nicht diagnostiziert werden [37]. Als Gründe hierfür werden mangelndes Bewusstsein und Kenntnis zu MODY und ungleichgewichtiger Zugang zu genetischer Testung genannt. Jeder/jede 8‑te im jugendlichen Erwachsenenalter diagnostizierte Diabetesfall (13 %) war nicht als klassischer Typ 1 oder Typ 2 Diabetes einzuordnen, demnach ein MODY anzunehmen [40]. Wird MODY nicht als solcher erkannt, resultiert häufig eine Fehlbehandlung [38].

Von den derzeit bekannten MODY Genen betrifft die überwiegende Mehrzahl (etwa 95 %) aller Fälle Mutationen in lediglich drei Genen, nämlich HNF/hepatic nuclear factor 1A, HNF/hepatic nuclear factor 4A und Glukokinase (GCK) [36, 41]. Die Kenntnis des Vorgehens bei Vorliegen einer dieser drei Mutationen ist somit für klinische Belange überaus bedeutsam.

#### Präzisionsmedizin

Der MODY – monogenetische Diabetes hat in den letzten Jahren auch im Kontext der Präzisionsmedizin an Aufmerksamkeit gewonnen, verstanden als maßgeschneiderte, individuelle Therapie für Patient:innen mit Diabetes, möglichst basierend auf der dem Diabetes zugrundeliegenden Pathophysiologie [42]. Die häufigsten heute bekannten MODY Formen beruhen entweder auf Mutationen von B‑Zell Transkriptionsfaktoren (HNF 1A und HNF 4A), wodurch die Insulinsekretion kompromittiert wird, oder auf einer Pathologie des die Freisetzung von Insulin auslösenden Glukoseschwellwertes (GCK). Aus den jeweiligen Mutationen resultierende Phänotypen sind klinisch ausreichend gut beschrieben und erlauben somit eine differential-therapeutische Zuordnung der Betroffenen [36]. Dies ist umso wichtiger, als die Möglichkeiten der Pharmakotherapie des Diabetes in den letzten Jahren deutlich zugenommen haben, wodurch die individuelle Therapieentscheidung generell deutlich komplexer geworden ist.

#### Diagnostik

Die Verdachtsdiagnose MODY erfolgt auf der Grundlage klinischer Kriterien (siehe Infobox „MODY/monogenetischer Diabetes: Klinische Charakteristika“). Aufgrund des autosomal-dominanten Erbganges sind Diabetesfälle in der Familie zu erwarten, insbesondere bei erstgradigen Verwandten, die oft über mehrere Generationen rückverfolgbar sind. Die Diagnose erfolgt oft bereits im Kindes- und jungen Erwachsenenalter. Eine Diabetesdiagnose vor Vollendung des 30. Lebensjahres ohne die Notwendigkeit einer Insulintherapie (Differentialdiagnose Typ 1 Diabetes), sollte an einen MODY Diabetes denken lassen. Phänotypisch handelt es sich in der Regel um normalgewichtige Patient:innen (Differentialdiagnose Typ 2 Diabetes).

##### MODY/monogenetischer Diabetes: Klinische Charakteristika. (Adaptiert nach [36])

- Nachweis einer transienten, neonatalen hyperinsulinämischen Hypoglykämie (HNF-4A)
- Familienanamnese, dem autosomal dominanten Erbgang entsprechend. Davon zu unterscheiden:
  - Der Typ 1 Diabetes tritt oft sporadisch auf; (lediglich) 2–6 % der Betroffenen haben einen ebenso betroffenen Elternteil
  - Der Typ 2 Diabetes tritt zwar auch familiär gehäuft auf, was allerdings dem gemeinsamen Lebensstil als auch gemeinsamen Diabetes Risiko Allelen geschuldet ist
- Frühmanifestierter Diabetes (vor dem 35 Lebensjahr; vor dem 25 Lebensjahr mit höherer Wahrscheinlichkeit)
- Nicht typisch für einen Typ 1 Diabetes
  - Pankreatische Autoantikörper nicht nachweisbar
  - Bei Insulintherapie geringe Insulindosis (zB < 0,5 IE/kgKG/d)
  - C‑Peptid nachweisbar (> 0,6 ng/ml) auch nach längerem Krankheitsverlauf (3–5 Jahre)
  - Keine Ketoazidose
- Nicht typisch für einen Typ 2 Diabetes
  - Normaler BMI (nicht übergewichtig oder adipös)
  - Keine Zeichen eines metabolischen Syndroms (normale Triglyceride, normales oder hohes HDL-Cholesterin)
  - Keine Zeichen einer *Acanthosis nigricans*
- Milde, stabile Hyperglykämie ohne Zeichen einer Progression (GCK)
- Gutes Ansprechen auf Sulfonlyharnstoffe

Das differentialdiagnostische Prozedere umfasst die Bestimmung der bei Autoimmundiabetes nachweisbaren Antikörper. Der Nachweis von Autoantikörpern macht das Vorliegen eines MODY sehr unwahrscheinlich, wenngleich Fälle eines koexistenten Typ 1 Diabetes beschrieben sind [43]. Ein MODY *clinical risk calculator* (University of Exeter, UK) ist online verfügbar und errechnet die Wahrscheinlichkeit für das Vorliegen eines MODY unter Berücksichtigung klinischer Parameter zur Selektion von Patient:innen für die molekulargenetische Diagnostik [44].

Die Diagnose MODY kann – gewissermaßen retrospektiv – auch nach langem Diabetesverlauf gestellt werden. Die korrekte Diabetes (Differential‑)Diagnose kann einerseits für Betroffene klinisch und differentialtherapeutisch (insbesondere gegenüber dem „klassischen“ Typ 2 Diabetes) relevant sein, andererseits auch für die direkte Nachkommenschaft im Sinne des autosomal-dominanten Erbganges.

Bei klinischem Verdacht auf das Vorliegen eines MODY erfolgt die Diagnosesicherung mittels Genotypisierung. Diese erfolgt für klinische Belange unter Verwendung kommerziell verfügbarer NGS (new generation sequencing) Plattformen. Diese ermöglichen die simultane und demnach mittlerweile vergleichsweise kostengünstige DNA Analyse bekannter MODY-assoziierter Gene [45].

#### Therapie

Für praktische Belange empfiehlt sich die Unterscheidung zwischen MODY Formen, die einerseits Mutationen Beta‑Zell-spezifischer Transkriptionsproteine (HNF) betreffen, sowie andererseits Mutationen im Glukokinase (GCK) Gen.

### HNF Mutationen: Erstlinientherapie

Sulfonlyharnstoffe werden bei HNF MODY Formen generell als Erstlinientherapie empfohlen [34, 36, 41, 42].

#### HNF1A MODY

Bei Patient:innen mit HNF1A MODY war der Sulfonylharnstoff Gliclazid um den Faktor 5 wirksamer als Metformin, und um den Faktor 4 wirksamer als bei klassischem Typ 2 Diabetes [46]. Selbst bei Patient:innen, die bereits über lange Zeit (Median 20 Jahre) eine Insulintherapie erhalten hatten (in einer Dosierung von Median 0,5 IE/kgKG/d) erbrachte die Umstellung auf 80 mg/Tag Gliclazid eine HbA1c Reduktion von 0,8 % [47]. Aufgrund der erhöhten Sensibilität der Beta‑Zellen auf die Wirkung der Sulfonylharnstoffe bei erhaltener (hoher) Insulinsensitivität, wird eine kleine Startdosis empfohlen. Diese reicht oft aus um den gewünschten Therapieeffekt zu erzielen; das Risiko von Hypoglykämien ist vermutlich höher als beim klassischen Typ 2 Diabetes. Die Gabe eines Glinides führte zu abgeschwächten postprandialen Blutzuckerspitzen und zu einem geringeren Risiko von Hypoglykämien, verglichen mit Glibenclamid [48].

#### HNF4A MODY

Das Ansprechen auf Sulfonlyharnstoffe ist bei HNF4A Mutationen geringer ausgeprägt als bei HNF1A MODY, sodass hier eher mit einem Beta‑Zellversagen und dann notwendiger Einleitung einer Insulintherapie zu rechnen ist [34].

### HNF Mutationen: Zweitlinientherapie und experimentelle Ansätze

Bei langer Krankheitsdauer kann es auch bei MODY zur nachlassenden Wirksamkeit der Sulfonlyharnstoffe kommen. Es wird angenommen, dass die Glukose-induzierte pankreatische Insulinsekretion ungeachtet fortgesetzter Sulfonylharnstoff Gabe mit einer Rate zwischen 1 und 4 % pro Jahr abfällt [34, 36]. Bei vielen Patient:innen mit HNF MODY wird daher nach langem Verlauf die Einleitung einer Insulintherapie erforderlich sein, insbesondere bei HNF4A Mutationen.

#### Inkretin-basierte Therapien

Klinische Studien haben jüngst die Wirksamkeit von DPP-IV Hemmern und GLP‑1 Rezeptoragonisten bei MODY (HNF1A) untersucht, mit dem Ziel die Beta‑Zellfunktion über einen längeren Zeitraum zu erhalten [49, 50]. Die Gabe von Linagliptin zu Glimepirid verbesserte bei 19 Patient:innen mit MODY HNF1A gegenüber Placebo die Glukosevariabilität als auch den mittleren Blutzucker (HbA1c minus 0,5 % bei einem Ausgangswert von 7,4 %), ohne dabei das Risiko von Hypoglykämien zu erhöhen [51]. Weiters war bei 16 Patient:innen der GLP‑1 Rezeptoragonist Liraglutid dem Sulfonlyharnstoff Glimeprid weitgehend ebenbürtig hinsichtlich glykämischer Kontrolle, bei geringerem Risiko von (milden) Hypoglykämien [52].

#### SGLT-2 Hemmer

Der Einsatz von SGLT‑2 Hemmern wird bei MODY nicht empfohlen [36]; das Risiko von Komplikationen, wie höhergradige Dehydratation und DKA (diabetische Ketoazidose) [53] wird nach heutigem Wissensstand höher gewertet als der potenzielle Nutzen. Die kombinierte Gabe von Insulin mit SGLT‑2 Hemmern wäre theoretisch denkbar, doch gibt es hierzu bislang keine Daten.

### Glucokinase (GCK) Mutationen

Glucokinase Mutationen stellen mit einer Häufigkeit von 0,1 % in der Allgemeinbevölkerung die häufigste MODY Form dar [54]. Betroffene weisen typischerweise milde, stabile Erhöhungen des Nüchternblutzuckers auf. HbA1c Werte können erhöht sein, typischerweise im Bereich unter 7,5 % [41]. GCK MODY wird bei Erwachsenen oft fälschlicherweise als Typ 2 Diabetes klassifiziert, im jüngeren Lebensalter folgt die Diagnose Diabetes häufig einer aus anderen Gründen erfolgten Blutanalyse im Sinne eines Zufallsbefundes. Mit der weiteren Verbreitung von Diabetes Früherkennungsprogrammen (wie etwa die kürzlich in Österreich erfolgte Kostenerstattung der HbA1c Messung) ist damit zu rechnen, dass GCK Mutationen häufiger detektiert werden. Daher ist die Kenntnis des GCK MODY von großer Bedeutung; ebenso die korrekte differentialdiagnostische Zuordnung (inklusive Genotypisierung), einerseits um Betroffene entsprechend aufklären zu können (hinsichtlich Erbgang, Krankheitsverlauf, Implikationen für die medikamentöse Behandlung, Implikationen für etwaige Schwangerschaften), andererseits um unnotwendige Pharmakotherapien hintanzustellen.

Patient:innen mit GCK MODY bedürfen prinzipiell *keiner* spezifischen, Blutzucker-senkenden Therapie [34, 36, 41, 42, 55], es sei denn es liegt konkomitant ein Typ 1/Autoimmundiabetes [43] oder ein Typ 2 Diabetes vor, oder im Falle einer Schwangerschaft (s. unten). Diese Empfehlung beruht auf Langzeitstudien, wonach Betroffene – wohl aufgrund der persistierend lediglich milden Hyperglykämie – keine diabetes-typischen Komplikationen entwickeln [55–58].

#### Schwangerschaft bei GCK MODY

Bei Trägerinnen einer GCK Mutation kann bei Eintreten einer Schwangerschaft regelhaft ein Gestationsdiabetes diagnostiziert werden; sei es, weil ein GCK MODY bereits vorbekannt ist, sei es, weil ein durchgeführter Glukose Belastungstest (oGTT) eine Pathologie ausweist (etwa einen erhöhten Nüchtern-Blutzucker). Die Betreuung von Schwangeren mit GCK MODY, insbesondere die Notwendigkeit der Gabe von Insulin hängt davon ab, ob der Fetus ebenfalls Träger der Mutation ist oder nicht [36, 59, 60]. Dies ist aus praktischen Belangen am ehesten aus der fetalen Biometrie abzuleiten. Wenn die Variante beim Fetus nicht vorliegt (oder der Verlauf einen solchen Umstand nahelegt), besteht ein hohes Makrosomie Risiko, woraus sich die Indikation zur Insulintherapie ableiten kann. Dabei sind oft vergleichsweise hohe therapeutische Insulindosen erforderlich, um maternale Blutzuckerzielwerte zu erreichen.

#### Seltenere MODY Formen

Es sind derzeit Mutationen in mehr als 15 MODY Genen beschrieben [36], wobei die Liste eine ständige Erweiterung erfährt. Oft erleichtert auch bei diesen selteneren Formen die der Mutation folgende Pathologie die differentialtherapeutische Zuordnung; häufig ist (analog den HNF Mutationen) die Beta‑Zelle betroffen und Sulfonlyharnstoffe gelten demnach als Therapie der ersten Wahl. Allerdings ist in der Interpretation von Molekulargenetischen-Befunden zu beachten, dass für manche (eben seltene) Mutationen, die im Zusammenhang mit MODY genannt werden, die tatsächliche, klinisch relevante Pathogenität noch der Klärung bedarf. Dies gilt etwa für Mutationen von BLK, KLF11, NEUROD1, PAX4, PDX1, die jeweils in der Allgemeinheit so häufig auftreten, dass der Zusammenhang mit MODY nicht gesichert ist [36]. Bei Diagnose einer dieser seltenen MODY Formen wird die Zuweisung an spezialisierte Zentren prinzipiell sinnvoll sein.

### Mitochondriale Diabetesformen

Mitochondriale Diabetesformen sind – sofern es sich um keine Spontanmutation handelt – maternal vererbt und häufig mit neuromuskulären Symptomen sowie Hörstörungen verbunden, wobei letztere häufig der Diabetesmanifestation vorausgehen. Laut Diabetes-Patienten-Verlaufsdokumentation (DPV)-Register machen diese Formen weniger als 1 % aller Diabetesfälle im Erwachsenenalter aus [61]. Den beiden Syndromen „maternally inherited diabetes and deafness“ (MIDD) und „mitochondrial encephalomyopathy with lactic acidosis and stroke-like episodes“ (MELAS) liegt die gleiche Genmutation zugrunde [62], vermutlich aufgrund der verschieden ausgeprägten Heteroplasmie kommt es jedoch zu unterschiedlicher Symptomatik [63]. Das DIDMOAD- oder Wolfram-Syndrom ist charakterisiert durch das Auftreten eines Diabetes insipidus, eines Diabetes mellitus, einer Optikusatrophie und Taubheit. Eine spezifische Therapie der mitochondrialen Diabetesformen existiert nicht, beim MELAS Syndrom werden L‑Arginin und auch Carnitin bzw. Coenzym Q10 zur Therapie der neurologischen Symptomatik versuchsweise eingesetzt [64].

### Neonataler Diabetes

Neonataler Diabetes ist definiert als Diabetesmanifestation vor dem 6. Lebensmonat, wobei transiente von permanenten Verläufen unterschieden werden. Ein Diabetes-Relaps bei transienten Verlaufsformen wird in bis zu 50 % der Fälle beschrieben [65]. In Österreich beläuft sich die Inzidenz auf 1/230.000 für transiente und 1/530.000 für permanente neonatale Diabetesformen [66]. Die Ursache ist meist monogenetisch, wobei 6q24 Abnormitäten (uniparentale Disomie, Duplikation, Methylierungsdefekte), Mutationen an Untereinheiten des ATP abhängigen Kalium-Kanals (*ABCC8, KCNJ11*) sowie Mutationen am Proinsulin codierenden *INS*-Gen, Glukokinase-Gen *GCK, HNF1β* ursächlich beschrieben wurden [67]. Neonataler Diabetes kann auch im Rahmen syndromaler Erkrankungen auftreten, wobei IPEX-Syndrom (*FOXP3*), Wolcott-Rallison-Syndrom (*EIF2AK3*), Pankreasagenesie (*PDX1, PTF1A*) ca. 10 % der neonatalen Diabetesfälle erklären. Die genetische Abklärung ist aufgrund therapeutischer Konsequenzen wichtig [68, 69]. Die initiale Behandlung mit Insulin unter Vermeidung von Hypoglykämien ist immer indiziert, wobei die Verwendung einer Insulinpumpe zur Applikation der geringen Insulinmengen bevorzugt Einsatz finden sollte. Eine sensorunterstütze Pumpentherapie ist bei ausreichend subkutanem Fettgewebe ab einem Körpergewicht von ca. 3 kg möglich. Eine orale Behandlung mit Sulfonylharnstoffen ist indiziert, wenn Mutationen an den Untereinheiten SUR1 und Kir6.2 des ATP abhängigen Kaliumkanals als Ursache für den vorliegenden neonatalen Diabetes genetisch bestätigt wurden [70]. Hier stehen auch orale Suspensionen zur Therapie bei Säuglingen zur Verfügung [71].

## Pankreopriver Diabetes

Generell kann jede Erkrankung, die zu einer diffusen Zerstörung des Pankreasgewebes führt, mit einem pankreopriven Diabetes einhergehen. In westlichen Ländern sind pankreoprive Ursachen für etwa 5–10 % aller Diabeteserkrankungen verantwortlich [72]. Ätiologisch kommen dafür traumatische oder infektiöse Ursachen ebenso wie Pankreasoperationen, Pankreasagenesie, Pankreaskarzinome oder Pankreatitiden in Frage. Letztere sind für etwa ¾ aller pankreopriven Diabetesformen verantwortlich [73]. Pankreaskarzinome stellen insofern eine Ausnahme dar, als sie auch bei lokalisiertem Vorliegen ohne diffuse Zerstörung des Pankreasgewebes zu einer diabetogenen Stoffwechsellage führen können, der Grund dafür ist bisher nicht klar [74]. Hereditäre Stoffwechselerkrankungen wie Zystische Fibrose und Hämochromatose können ebenso zu pankreoprivem Diabetes führen. Auch die seltene und primär in asiatischen Ländern vorkommende fibrokalkulöse Pankreatopathie (FCPP) kann sekundär einen Diabetes mellitus bedingen [1, 55].

Bei postoperativen Patient:innen ist das Risiko für die Entwicklung eines pankreopriven Diabetes abhängig von der Art der Operation. Naturgemäß beträgt das Risiko 100 % bei total pankreatektomierten Patient:innen, bei Patient:innen mit Whipple Operation (partielle Pankreatikoduodenektomie) wird das Risiko in älteren Studien mit 26 % beziffert, wobei ein beträchtlicher Teil der damals untersuchten Patient:innen schon präoperativ an einem Diabetes erkrankt war [75].

Diagnostische Kriterien eines pankreopriven Diabetes umfassen eine Betazelldysfunktion, das Fehlen von Autoimmunantikörper und das Vorliegen einer Erkrankung des exokrinen Pankreas (Diagnostik s. unten) [76, 77]. Die betroffenen Patient:innen weisen typischerweise eine hohe Insulinsensitivität auf, vorausgesetzt, dass dem pankreoprivem Diabetes kein Diabetes mellitus Typ 2 oder Prädiabetes mit Insulinresistenz vorausgegangen ist.

Eine zufriedenstellende glykämische Kontrolle ohne Auftreten von klinisch signifikanten Hypoglykämien kann sich bei pankreoprivem Diabetes als schwierig herausstellen. Ursache dafür sind das Fehlen der gegenregulatorisch wirkenden Hormone Somatostatin und Glukagon ebenso wie eine durch die Grundkrankheit bedingte Malabsorption. Hinzu können schlechte Compliance und Lifestyle-Problematik bei Patient:innen mit alkoholischer Pankreatitis kommen [76].

Die systemische Insulinsensitivität ist bei pankreoprivem Diabetes typischerweise normal oder sogar erhöht, die hepatische Insulinsensitivität kann auch vermindert sein. Zur Therapie des pankreopriven Diabetes liegen keine spezifischen internationalen Richtlinien vor. Eine Metformintherapie kann bei gleichzeitig bestehender Insulinresistenz zu einer Verbesserung der Glykämie führen, allerdings wird Metformin aufgrund der gastrointestinalen Nebenwirkungen von diesem Patient:innenkollektiv meist schlecht toleriert. Bei hoher Insulinsensitivität ist der Effekt auf die Glykämie zudem als begrenzt anzunehmen, allerdings belegen Studien ein deutlich vermindertes Pankreaskarzinomrisiko unter Metformintherapie [77]. Inkretin-basierte Therapien sollten aufgrund eines potenziell erhöhten Pankreatitisrisikos bis zur endgültigen Klärung der Sicherheit der Wirkstoffe primär nicht zur Therapie eines pankreopriven Diabetes herangezogen werden. Sulfonylharnstoffe oder Glinide sind grundsätzlich geeignet, die Insulinsekretion bei vorhandener Betazellrestfunktion zu steigern, der Einsatz von Sulfonylharnstoffen ist allerdings aufgrund des besonderen Hypoglykämierisikos dieser Patient:innen streng zu monitieren. SGLT-2-Hemmer sind aufgrund des vorliegenden absoluten Insulinmangels und des damit verbunden erhöhten Risikos einer euglykämischen Ketoazidose nicht zu empfehlen. Therapie der Wahl ist daher bei einem Großteil der betroffenen Patient:innen Insulin, optimalerweise in Form eines Basis-Bolus-Regimes.

### Diabetes bei zystischer Fibrose (CFRD)

Diabetes mellitus bei zystischer Fibrose (engl. cystic fibrosis related diabetes, CFRD) stellt die häufigste extra-pulmonale Komplikation bei Patient:innen mit zystischer Fibrose dar [78]. Die Prävalenz steigt mit zunehmendem Patient:innenalter und liegt bei Erwachsenen bei ca. 50 % [78], bei Patient:innen nach Organtransplantation (Lungen- oder Lebertransplantation) vermutlich noch höher.

Pathophysiologisch steht vor allem die verminderte Insulinsekretion durch die krankheitsspezifische Pankreasfibrose im Vordergrund. Einige Autor:innen beschreiben zudem eine variable Insulinresistenz durch rezidivierende Infekte und wiederholte Glukokortikoidtherapie im Rahmen von Infektexazerbationen [78, 79]. Der CFRD ist assoziiert mit einer Verschlechterung der Lungenfunktion, des Ernährungsstatus sowie letztendlich des Überlebens [80].

Als Screening-Methode wird die Durchführung eines oralen Glukosetoleranztests jährlich ab dem 10. Lebensjahr unter stabilen klinischen Bedingungen empfohlen [78, 81]. Zusätzlich wird ein Screening in bestimmten klinischen Situationen empfohlen: während eines stationären Aufenthalts aufgrund einer Infektexazerbation (Erstellung eines Glukoseprofils mit Nüchtern- und postprandialen Werten), bei enteraler Ernährung sowie bei Frauen mit Kinderwunsch vor der geplanten Schwangerschaft, zwischen der 11. und 14. sowie zwischen der 24. und 28. Schwangerschaftswoche, und 6–12 Wochen nach der Entbindung.

Als Grenzwerte für die Diagnose eines CFRD gelten die allgemeinen Grenzwerte zur Diabetes-Diagnose. Zudem wurde wiederholt ein erhöhter 1‑Stunden postprandialer Wert > 200 mg/dl mit einer klinischen Verschlechterung in Zusammenhang gebracht, weswegen bei Patient:innen mit dieser Form der Glukoseintoleranz (indeterminate glucose tolerance (INDET)) in Situationen klinischer Verschlechterung eine vorübergehende Insulintherapie in Erwägung gezogen werden sollte.

HbA1_C_ mit einem Cut-Off von 5,5 %, das kontinuierliche Glukosemonitoringsystems (CGMS) oder der HOMA-B-Index werden aktuell in Studien evaluiert, können derzeit als Screeningmethoden jedoch noch nicht empfohlen werden [55]. Der Einsatz des CGMS wurde in einigen Studien empfohlen, ist aber als generelle Screening-Methode noch nicht etabliert. Sie sollte jedoch bei Patient:innen mit klinischer Verschlechterung und Hinweis auf CFRD, jedoch normalem OGTT, eingesetzt werden.

Die Therapie der Wahl ist jegliche Insulintherapie, wobei sämtliche Therapieformen in Frage kommen: Der Insulinbedarf ist meist niedriger als bei Patient:innen mit Typ 1 Diabetes und wird mit etwa 0,4 IE/kg/Tag bei Jugendlichen, 0,5 IE/kg/Tag bei Erwachsenen und 0,6 IE/kg/Tag bei Patient:innen nach Organtransplantation angegeben [82, 83]. Studien mit oralen Antidiabetika, insbesondere Sulfonylharnstoffen, konnten nur einen vorübergehenden Effekt zeigen und werden derzeit nicht empfohlen. Ziel der Insulintherapie ist vor allem das Erreichen eines anabolen Stoffwechsels um die Versorgung mit Makronährstoffen und Aufrechterhaltung des Körpergewichts zu sichern [55]. Hinsichtlich diätischer Empfehlungen unterscheidet sich der CFRD wesentlich von anderen Diabetesformen, da sowohl von einer Kalorien‑, Kohlenhydrat-, und Protein-reduzierten Diät abgeraten und eine Protein- und Salz-reiche Ernährung sogar empfohlen wird [81]. Lediglich „Softdrinks“ sollen in der Ernährung weggelassen werden, da sie zu teils schwer beherrschbaren Blutzuckeranstiegen führen [84].

Die Verlaufskontrolle unterscheidet sich wesentlich von anderen Diabetesformen. Als primärer Parameter gilt der Gewichtsverlauf. Jeder ungewollte Gewichtsverlust bei Patient:innen mit CFRD soll Anlass für eine Therapiekontrolle geben. Ergänzend kann das HbA1C herangezogen werden, wobei ein HbA1_C_ im Normbereich, also < 6 %, zumindest < 6,5 %, angestrebt werden sollte.

Neben den bereits beschriebenen Komplikationen des CFRD (Gewichtsverlust, Verschlechterung des Ernährungszustandes und der Lungenfunktion) spielen von den klassischen diabetischen Spätkomplikationen vor allem mikroangio- und neuropathische Komplikationen eine Rolle, die aufgrund der in den letzten Jahrzehnten deutlich gesteigerten Lebenserwartung bei CF zunehmen werden. Deshalb wird ein jährliches Screening beginnend 5 Jahre nach Diabetes-Manifestation empfohlen. Akutkomplikationen wie Hypoglykämien und diabetische Ketoazidose werden beim CFRD relativ selten beobachtet, letztere kommt aufgrund des meist nur relativen Insulinmangels so gut wie nie vor.

## Andere Diabetesformen

Diese seltenen Erkrankungen umfassen infektiöse Formen (kongenitale Röteln) oder auch das Stiffman-Syndrom (auch Stiff-Person-Syndrom genannt). Beim letzteren handelt es sich um eine Autoimmunerkrankung mit neurologischer Symptomatik, die spontan oder paraneoplastisch auftreten kann.

## Exokrine Pankreasinsuffizienz (EPI)

### Prävalenz und Pathogenese

In der Literatur werden EPI-Prävalenzen zwischen 10 und 56 % bei Patient:innen mit Typ 1 Diabetes angegeben [85–89]. Eine schwere EPI, die sich definiert durch eine Elastase-1-Konzentration im Stuhl < 100 µg/g, wurde dabei bei 10–30 % der Patient:innen festgestellt. Eine EPI ist bei jedem dritten Patienten/jeder dritten Patientin mit Typ 2 Diabetes beschrieben, wobei mehr als die Hälfte eine schwere Form aufweist [85, 86, 89–91]. Ursächlich findet sich bei DM1 eine verminderte Dichte an parasympathischen Axonen im exokrinen Pankreas [92], zudem kommt es im Rahmen der Entzündung auch zu einer Störung der Betazellregeneration, was aufgrund des gemeinsamen Ursprungs von exokrinen und endokrinen pankreatischen Vorläuferzellen ebenso zur exokrinen Insuffizienz beiträgt [93–95]. Die Pathophysiologie von EPI bei Typ 1 und Typ 2 Diabetes ist nicht vollständig geklärt, inkludiert aber Inflammation, Fibrose und Steatose des exokrinen Pankreas und Störungen in der Insel-Azinar-Achse [96].

In einer rezenten Studie bei Patient:innen mit Typ 1 und Typ 2 Diabetes wurde eine EPI, definiert als eine fäkale Elastase 1 Konzentration < 200 µg/g, bei 13 % nachgewiesen [97]. In dieser Studie war EPI häufiger bei Typ 1 vs Typ 2 DM und die Diabetesdauer ein Risikofaktor für EPI. In anderen Studien wurde keine Assoziation zwischen Diabetesdauer und EPI gefunden [87]. In einer Metanalyse von 17 Studien mit 3662 Menschen mit Diabetes war eine EPI, definiert als fäkale Elastase 1 < 200 µg/g ebenfalls häufiger bei Typ 1 (38,62 %) als Typ 2 DM (28,12 %) [98].

Die chronische Pankreatitis ist die häufigste Ursache der EPI bei Erwachsenen [99, 100]. EPI ist ein unabhängiger Risikofaktor für Mortalität bei chronischer Pankreatitis [101]. Etwa 85 % der Neugeborenen mit cystischer Fibrose (CF) sind pankreatisch insuffizient (PI) [102]. Neben Pankreasresektionen [103] können auch Magen- oder Dünndarmresektionen durch den Verlust der Sekretin- und Cholecystokinin Synthese bzw. rascher Magenentleerung mit EPI assoziiert sein, woran auch bei Patient:innen nach bariatrischer Chirurgie gedacht werden sollte [104]. Eine altersbedingte Pankreasatrophie (5 % ab > 70 Jahren, 10 % > 80 Jahren) kann ebenfalls mit EPI einhergehen [105]. Andere seltene Ursachen einer EPI sind die hereditäre Hämochromatose, Gastrinome (Inaktivierung von Pankreasenzymen durch Magensäure) und die Zöliakie.

### Klinische Manifestationen bei EPI

Patient:innen mit milder EPI können asymptomatisch sein oder über leichtes abdominelles Unbehagen und Blähungen mit normalem Stuhlgang berichten. Bei schwerer EPI kann es infolge von Fett- und Protein-Maldigestion zu Gewichtsverlust kommen. Eine offenkundige Steatorrhoe tritt erst bei Verlust von ca. 90 % der glandulären Funktion auf und geht mit übelriechenden, fettigen Stühlen mit reduzierter Konsistenz, die sich schwer wegspülen lassen, einher. Weitere Symptome sind Blähungen, abdominelle Krämpfe, Flatulenz. Obwohl klinisch symptomatische Vitaminmangelzustände mit metabolischer Knochenerkrankung oder gestörter Nachtsicht selten sind, sollte ein Mangel an fettlöslichen Vitaminen (ADEK) ausgeschlossen werden [100, 106–108]. Seltener liegt ein Vitamin B12 Mangel vor (reduzierter intestinaler pH).

### Screening und Diagnostik der exokrinen Pankreasinsuffizienz

Wegweisend ist hierzu die Anamnese. EPI sollte bei Patient:innen mit chronischer Diarrhoe/Steatorrhoe und chronischen Abdominalschmerzen, aber auch bei Patient:innen mit milderen Symptomen wie Blähungen und typischer Bildgebung für chronische Pankreatitis (z. B. Kalzifikationen und/oder Pankreasgangdilatationen und/oder Pseudozysten im Ultraschall, CT oder MRT) oder Pankreasatrophie suspiziert werden.

Bei entsprechender Symptomatik folgt zur weiteren Diagnostik meist ein indirekter (einfach, günstig) oder direkter Pankreas-Funktionstest (Stimulation des Pankreas durch hormonelle Sekretagoga mit nachfolgender Entnahme und Analyse von Duodenalflüssigkeit, z. B. Sekretin Test: aufwendig, invasiv, schlechte Patiententoleranz). Die Bestimmung der Elastase-1-Konzentration im Stuhl mittels Immunassay (indirekter Test) gilt als Standardtest mit einer Sensitivität von etwa 65 % bei milder und bis zu 100 % bei schwerer Form [109]. Eine fäkale Elastase-1 < 200 µg/g gilt als pathologisch, eine fäkale Elastase-1 < 100 µg/g gilt als schwere EPI.

Aufgrund der relativ hohen Prävalenz der EPI ist bei gastrointestinaler Beschwerdesymptomatik ein Screening bei Patient:innen mit Diabetes sinnvoll [110–113].

Die differentialdiagnostisch in Erwägung zu ziehenden Erkrankungen ergeben sich aus der genannten Symptomatik der EPI und umfassen insbesondere die zahlreichen anderen Ursachen einer chronischen Diarrhoe. Speziell sind eine autonome Neuropathie des Magen-Darm-Traktes sowie gastrointestinale Nebenwirkungen oraler Antidiabetika (Metformin, Acarbose, Inkretinanaloga) zu erwägen. Auch Zuckeraustauschstoffe, wie die häufig verwendete Fruktose oder Sorbit, können bei Unverträglichkeit vergleichbare Symptome verursachen. Differentialdiagnostisch kommt auch eine häufig bei Menschen mit Diabetes vorliegende bakterielle Fehlbesiedlung im Dünndarm in Betracht [114].

Speziell bei Patient:innen mit Typ 1 Diabetes sollte an eine Zöliakie gedacht und diese mittels serologischer Diagnostik (endomysiale Antikörper [EMA], Antikörper gegen Gewebstransglutaminase [tTG] oder deamidiertes Gliadin Peptid [DGP]) ausgeschlossen werden. Ein normales Stuhl Calprotectin ist bei der Unterscheidung zwischen organischen/entzündlichen (z. B. M. Crohn, Colitis ulcerosa) und funktionellen gastrointestinalen Erkrankungen (z. B. Reizdarm, funktionelle Dyspepsie) hilfreich. Zusätzlich ist der Ausschluss eines Pankreaskarzinoms (mittels CT/MRT und/oder Endosonographie) wichtig, welches bei Diabetes im Vergleich zur nicht diabetischen Bevölkerung häufiger auftritt [74].

### Therapie

Die Therapie der EPI besteht in einer dem Ausmaß der EPI sowie der Ernährung angepassten, ausreichenden Substitution von Pankreasenzymen. Bei schwerer Pankreasinsuffizienz sind pro Gramm Fett 2000 bis 4000 Einheiten Lipase nötig. Die Dosisfindung richtet sich nach den klinischen Beschwerden des Patient:innen mit dem Ziel der Beschwerdefreiheit. Eine Ernährungsberatung zum möglichst guten Einschätzen der Fettmenge in der Nahrung und zur Vorbeugung von Mangelzuständen sollte zusätzlich erfolgen. Die Evidenz für die Behandlung der Malabsorption durch mangelnde Pankreasenzymsekretion ist sehr gut [107, 108, 110, 115–120]. Fettlösliche Vitamine sollten zusammen mit Pankreasenzymen eingenommen werden. Von Nikotinkonsum sollte unbedingt abgeraten werden, da Rauchen einen unabhängigen Risikofaktor für EPI sogar bei Patient:innen ohne vorhergehende Pankreaserkrankung zu sein scheint [121].

## Interessenkonflikt

S. Kaser hat von folgenden Unternehmen, die zT auch fördernde Mitglieder der ÖDG sind, Forschungsunterstützungen und/oder Honorare erhalten: AstraZeneca, Boehringer Ingelheim, Eli Lilly, Merck Sharp & Dohme, Novartis, Novo Nordisk, Viatris. S.E. Hofer hat in den letzten 3 Jahren von folgenden Unternehmen, die auch förderndes Mitglied der ÖDG sind, Forschungsunterstützungen und/oder Honorare erhalten: Eli Lilly, Medtronic, Insulet, Dexcom und Ypsomed. L. Kazemi-Shirazi hat Vortragshonorare von MSD, AbbVie, Ferring, MerckSerono/Dr Falk, Chiesi, Novartis, Roche, Abbott, Phadia Austria/Thermo Fisher Scientific, CSL-Behring, Janssen-Cilag Pharma, Labors.at GmbH, Vertex, ENML Laborbetriebs GmbH und Kongressunterstützungen von: Mylan, Abbott, MSD, Gilead, MerckSerono/Dr Falk, Novartis, Pfizer, Janssen-Cilag Pharma, Chiesi, Shire, Abbvie, Takeda, Vertex erhalten. Konsulent/Berater-Tätigkeit: MSD, Takeda. Y. Winhofer hat von folgenden Unternehmen, die auch fördernde Mitglieder der ÖDG sind, Forschungsunterstützungen und/oder Honorare erhalten: Sanofi Aventis, AstraZeneca, Novartis, Eli Lilly. H. Sourij hat von folgenden Unternehmen, die auch fördernde Mitglieder der ÖDG sind, Forschungsunterstützungen und/oder Honorare erhalten: Amgen, AstraZeneca, Boehringer Ingelheim, Bayer, Daiichi Sankyo, Eli Lilly, Kapsch, Merck Sharp & Dohme, Novo Nordisk, Novartis, sanofi-aventis. H. Brath hat von folgenden Unternehmen, die auch fördernde Mitglieder der ÖDG sind, Forschungsunterstützungen und/oder Honorare erhalten: Abbott Diabetes Care, Amgen, AstraZeneca, Boehringer Ingelheim, Eli Lilly, Medtronic, MedMedia, MSD, Novartis, Novo Nordisk, Sandoz, Sanofi, Servier. M. Riedl hat von folgenden Unternehmen, die auch fördernde Mitglieder der ÖDG sind, Forschungsunterstützungen und/oder Honorare erhalten: AstraZeneca, Boehringer Ingelheim, Eli Lilly, Novo Nordisk, Novartis, Medronic, sanofi-aventis und Takeda sowie vom Bürgermeisterfond der Stadt Wien Proj.Nr.: 9073. M. Resl hat von folgenden Unternehmen, die auch fördernde Mitglieder der ÖDG sind, Forschungsunterstützungen und/oder Honorare erhalten: Boehringer Ingelheim, Eli Lilly, Novo Nordisk, Viatris, Amgen. M. Clodi hat von folgenden Unternehmen, die auch fördernde Mitglieder der ÖDG sind, Forschungsunterstützungen und/oder Honorare erhalten: AstraZeneca, Boehringer Ingelheim, Eli Lilly, Merck Sharp & Dohme, Novartis, Novo Nordisk, sanofi-aventis, Roche. T. Stulnig hat von folgenden Unternehmen, die auch fördernde Mitglieder der ÖDG sind, Forschungsunterstützungen und/oder Honorare erhalten: AstraZeneca, Boehringer Ingelheim, Eli Lilly, Novartis, Novo Nordisk, Sanofi. C. Ress hat von folgenden Unternehmen, die zT auch fördernde Mitglieder der ÖDG sind Kongressunterstützungen und/oder Honorare erhalten: AstraZeneca, NovoNordisk, Pfizer, Boehringer Ingelheim, Amrypt, Sanofi. A. Festa und A. Luger geben an, dass kein Interessenkonflikt besteht.
